# Transcriptomic analysis of *Streptococcus agalactiae* periprosthetic joint infection

**DOI:** 10.1002/mbo3.1256

**Published:** 2021-12-17

**Authors:** Hye‐Kyung Cho, Thao Masters, Kerryl E. Greenwood‐Quaintance, Stephen Johnson, Patricio R. Jeraldo, Nicholas Chia, Meng Pu, Matthew P. Abdel, Robin Patel

**Affiliations:** ^1^ Division of Clinical Microbiology, Department of Laboratory Medicine and Pathology Mayo Clinic Rochester Minnesota USA; ^2^ Department of Health Sciences Research Mayo Clinic Rochester Minnesota USA; ^3^ Center for Individualized Medicine Mayo Clinic Rochester Minnesota USA; ^4^ Department of Surgery Mayo Clinic Rochester Minnesota USA; ^5^ Department of Medicine, Division of Gastroenterology and Hepatology Mayo Clinic Rochester Minnesota USA; ^6^ Department of Orthopedic Surgery Mayo Clinic Rochester Minnesota USA; ^7^ Division of Infectious Diseases, Department of Medicine Mayo Clinic Rochester Minnesota USA

**Keywords:** prosthesis‐related infections, RNA‐seq, *Streptococcus agalactiae*, transcriptome

## Abstract

Although *Streptococcus agalactiae* periprosthetic joint infection (PJI) is not as prevalent as staphylococcal PJI, invasive *S. agalactiae* infection is not uncommon. Here, RNA‐seq was used to perform transcriptomic analysis of *S. agalactiae* PJI using fluid derived from sonication of explanted arthroplasties of subjects with *S. agalactiae* PJI, with results compared to those of *S. agalactiae* strain NEM316 grown in vitro. A total of 227 genes with outlier expression were found (164 upregulated and 63 downregulated) between PJI sonicate fluid and in vitro conditions. Functional enrichment analysis showed genes involved in mobilome and inorganic ion transport and metabolism to be most enriched. Genes involved in nickel, copper, and zinc transport, were upregulated. Among known virulence factors, *cyl* operon genes, encoding β‐hemolysin/cytolysin, were consistently highly expressed in PJI *versus* in vitro. The data presented provide insight into *S. agalactiae* PJI pathogenesis and may be a resource for identification of novel PJI therapeutics or vaccines against invasive *S. agalactiae* infections.

## Graphical abstract

Transcriptomic analysis of *Streptococcus agalactiae* periprosthetic joint infection (PJI) was performed using fluid derived from sonication of explanted arthroplasties of subjects with *S. agalactiae* PJI. Results were compared to those of *S. agalactiae* strain NEM316 grown *in vitro*. Results of functional enrichment analysis are shown; one interesting finding was that genes involved in inorganic ion transport and metabolism were enriched in PJI.

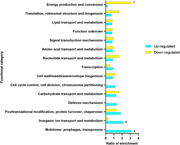

## 1 INTRODUCTION

Periprosthetic joint infection (PJI) causes significant morbidity and mortality, and healthcare cost burden (Bozic et al., 2010, 2009; Brochin et al., 2018; Kurtz et al., 2012; Lum et al., 2018; Natsuhara et al., 2019). *Staphylococcus aureus* and *Staphylococcus epidermidis* are the most frequent causes of PJI, causing ∼65% of cases (Zimmerli et al., 2004). However, other bacteria, including streptococci, enterococci, and Gram‐negative bacilli, also contribute to PJI; conceivably, each could be considered as causing a distinct disease state.

*Streptococcus agalactiae*, a component of the gastrointestinal microbiota also found in the genitourinary tract of some adults, is an important pathogen in newborns and pregnant women. Recently, the incidence of invasive *S. agalactiae* infections has been increasing in nonpregnant adults, particularly among those with comorbidities and older individuals (Edwards & Baker, 2005). While there is a difference in serotype distribution of *S. agalactiae* causing neonatal and adult diseases (Schuchat, 1998), other characteristics of the bacterium that might affect these two populations have not been elucidated.

Bone and joint infections, including osteomyelitis, spondylodiscitis, and native and periprosthetic joint infection, are common manifestations of *S. agalactiae* infections in adults (Corvec et al., 2011; Farley & Strasbaugh, 2001; Oppegaard et al., 2016). *S. agalactiae* is responsible for <10% of PJIs, most frequently “delayed” or “late‐onset” PJIs (Sendi et al., 2011; Tande & Patel, 2014). Infection is presumed to be hematogenous in most cases, with the gastrointestinal tract, genitourinary tract, and possibly skin being common sources (Tande & Patel, 2014; Triesenberg et al., 1992; Zeller, Lavigne, Leclerc et al., 2009). There are conflicting reports on the outcomes of *S. agalactiae* PJI. While some studies report remission rates of *S. agalactiae* PJI to be higher than those of staphylococcal PJI (Fiaux et al., 2016), others suggest that streptococcal PJIs as a whole have high treatment failure rates (Akgün et al., 2017), with *S. agalactiae* having worse outcomes than other *Streptococcus* species (Mahieu et al., 2019; Zeller, Lavigne, Biau et al., 2009); reasons behind this are unknown.

Understanding transcript profiles of bacteria under physiological or pathological conditions may help identify genomic elements that contribute to disease processes (Croucher & Thomson, 2010; Wang et al., 2009). Massive parallel sequencing can be used to analyze transcriptomes via complementary DNA (cDNA) sequencing—so‐called, RNA‐seq (Kukurba & Montgomery, 2015; Wang et al., 2009), providing all transcriptomic data in an unbiased manner and at a higher resolution than microarray or individual gene or gene panel analysis (Croucher & Thomson, 2010).

Here, a transcriptome study based on RNA‐seq analysis of in vivo *S. agalactiae* RNA from samples derived from sonication of explanted arthroplasties is presented. *S. agalactiae* PJI RNA‐seq data were compared to previously generated RNA‐seq data from *S. agalactiae* strain NEM316 grown in vitro (Rosinski‐Chupin et al., 2015), to explore PJI‐specific gene expression profiles.

## 2 MATERIALS AND METHODS

### 2.1 Materials

Sonicate fluid samples collected between April 2005 and August 2016 from six patients who underwent hip or knee arthroplasty revision for *S. agalactiae* PJI were studied. A publicly available RNA‐seq transcriptome data set from *S. agalactiae* NEM316 (a serotype III [ST‐23] reference strain from the blood of a neonate with early‐onset *S. agalactiae* disease [Glaser et al., 2002]) grown to mid‐exponential phase in Todd Hewitt medium (three replicates), was used to compare gene expression patterns with RNA‐seq data from sonicate fluid samples (BioProject accession number PRJEB8097: https://www.ncbi.nlm.nih.gov/bioproject/PRJEB8097 [BioSample accessions SAMEA3180396, SAMEA3180402, SAMEA3180416]). The six *S. agalactiae* isolates cultured from sonicate fluid were also used for pan‐genome construction.

### 2.2 Sample handling

Explanted prostheses were transported to the clinical microbiology laboratory in solid jars. Implant processing was performed according to an established clinical protocol that includes vortexing and sonication in Ringer's solution (Trampuz et al., 2007). Sonicate fluid samples were concentrated 100‐fold by centrifugation and immediately stored at −80°C without RNA an stabilizer until RNA was extracted and sequenced.

### 2.3 Bacterial whole genome sequencing and pan‐genome construction

*S. agalactiae* was identified per standard protocols in the Mayo Clinic Clinical Microbiology Laboratory. *S. agalactiae* isolates were designated 1–6 corresponding to their associated subject number. Genomic DNA was extracted from the six isolates cultured from PJI subjects using the Zymo Research Quick‐DNA Fungal/Bacterial Miniprep Kit (Zymo Research) and quantified using a Qubit 2.0 Fluorometer (Thermo Fisher Scientific). Sequencing libraries were prepared using a Nextera® XT PE Kit (Illumina Inc.). Sequencing was performed on an Illumina HiSeq 4000 with a 2 × 150‐base pair setting and 60 sample libraries multiplexed per flow cell.

Bacterial genomes were assembled from raw reads using a *de novo* assembler SKESA v2.4.0 (Souvorov et al., 2018) and annotated by Prokka v1.14.5 using the *S. agalactiae* 2603V/R genome as the reference genome for purposes of gene annotation (Seemann, 2014). A pan‐genome was constructed with Roary v3.13.0 using annotated fragmented *de novo* assemblies to identify core and accessory genes (Page et al., 2015). This pan‐genome served as a common reference for transcript quantification and outlier analysis between the RNA‐seq data from sonicate fluid and NEM316.

### 2.4 Phylogeny and virulence gene profiling

A phylogeny based on 1000 common gene families across the six isolates plus *S. agalactiae* strains NEM316 and 2603V/R was constructed using the CodonTree method at the Phylogenetic Tree Building Service from PATRIC Bioinformatics Resource Center (Davis et al., 2019). Phylogeny was midpoint rooted. Virulence gene content was profiled using the database of virulence factors of pathogenic bacteria (VFDB; Liu et al., 2019) through the interface at PATRIC.

### 2.5 Serotyping, multilocus sequence typing, and pilus typing

Artemis was used to annotate and extract capsular locus sequences from *S. agalactiae* isolate whole genome sequences (Rutherford et al., 2000). Extracted capsular locus sequences from each isolate were used to assign serotype, based on the highest identity using a BLAST query. The sequence type of each *S. agalactiae* isolate was determined by comparing allelic profiles of housekeeping genes *adhP*, *pheS*, *atr*, *glnA*, *sdhA*, *glcK*, and *tkt* (Jones et al., 2003) to the PubMLST *S. agalactiae* database (https://pubmlst.org/organisms/streptococcus-agalactiae) with SeqSphere+ software version 6.0.2 (Ridom GmbH). Pilus type was assigned by comparing allelic profiles of pilus genes (PI‐1, *sag0645–0650*; PI‐2a, *sag1404–1408*; PI‐2b, *sag2190–2194*) with a sequence query to the database.

### 2.6 RNA isolation and sequencing

RNA from sonicate fluid was isolated using the miRNeasy Serum/Plasma Kit (QIAGEN) and subjected to genomic DNA and bacterial ribosomal RNA (rRNA) removal using RNase‐free DNase I (QIAGEN) and the Ribo‐Zero rRNA Removal Kit (Bacteria; Illumina). Following purification using an RNeasy MinElute Cleanup Kit (QIAGEN), rRNA‐depleted RNA was eluted in 30 µl RNAse‐free water. RNA quantity and integrity were evaluated using a Qubit 2.0 Fluorometer coupled with a Qubit RNA High‐Sensitive Assay Kit (Thermo Fisher Scientific), and an Agilent 4200 TapeStation system (Agilent).

Next‐generation sequencing cDNA libraries were constructed using the Ovation SoLo RNA‐seq System (NuGEN Technologies) from 1 ng of input RNA, as previously described (Masters et al., 2018). External RNA Control Consortium RNA Spike‐In Mix 1 (Thermo Fisher Scientific) was used as a control to measure variability in the library generation process. Following cDNA synthesis and amplification, a SoLo AnyDeplete probe mix (NuGEN Technologies) was added to the libraries to deplete human rRNA sequences. The resulting cDNA libraries were sequenced on an Illumina HiSeq 4000 with 10 samples multiplexed per lane, producing 100‐base pair, paired‐end reads.

### 2.7 RNA‐seq analysis

Raw sequencing reads were analyzed to identify microbial RNA, as previously described (Thoendel et al., 2016), with minor modifications. RNA‐seq adapter sequences were trimmed with Atropos 1.1.19 (Didion et al., 2017), and human reads removed using BioBloom tools 2.1.1 (Chu et al., 2014). Taxonomy was assigned with Livermore Metagenomics Analysis Toolkit 1.2.6 using k‐mer identifiers and the kML+H.noprune.4‐14.2025.db database (Ames et al., 2013).

RNA‐seq reads were pseudoaligned to the pan‐genome constructed as described above (Tettelin et al., 2005) and transcript abundances were quantified using Kallisto version 0.42.4 (Bray et al., 2016) and converted to transcripts per million (TPM). For the external NEM316 data, data from the three replicates were aggregated by calculating the mean TPM. Outlier expression analysis was performed by calculating a modified *z*‐score for each gene *g*_ij_ in a sample *I* with *j* genes present in the core genome such that: *z* = [log_2_(*g*_ij_) − median(log_2_(*g*_i_)]/[1.4826 × MAD(log_2_(*g*_i_)], with a pseudocount added, if necessary. For this study, any gene with |*z*|>3 was considered an outlier.

### 2.8 Homology modeling of protein structure

To predict protein homology, protein structures of selected genes were generated from amino acid sequences derived from RNA‐seq data using a web‐based bioinformatics server, Phyre2 (Kelley et al., 2015). Predicted structural models were retrieved for selected sequences, queried and templated in Phyre2, with a representation of structures drawn using Chimera (Pettersen et al., 2004).

### 2.9 Statistical analysis

GraphPad Prism (ver. 8.0; GraphPad Software) was used to perform a Fisher's exact test to access functional enrichment of differentially expressed genes belonging to the specific clusters of orthologous genes (COGs) category.

## 3 RESULTS

### 3.1 Description of subjects

Six subjects with *S. agalactiae* PJI (mean age: 62 years, range: 42–73 years) who underwent surgery at Mayo Clinic from 2006 to 2016 were studied, four (67%) of whom were male. All had local pain at the involved site, with fevers and/or chills. Two had undergone hip and four knee arthroplasty. The age of implanted material at the time of surgery ranged from 29 days to 5.4 years, including two cases of “early” (less than 3 months after placement), 1 of “delayed” (3 months to 1–2 years after placement) and 3 of “late” (more than 1–2 years after implantation) infection (Tande & Patel, 2014). *S. agalactiae* was isolated from sonicate fluid culture from all subjects (Table 1). There was no obvious coinfection.

**Table 1:** Characteristics of six subjects with *Streptococcus agalactiae* periprosthetic joint infection

Subject	Isolate number	Year	Age (years)	Sex	Site of implant	Implant age at revision	Duration of symptoms (days)	*S. agalactiae* isolated from		Preoperative antibiotic treatment	Surgical procedure	Serotype	MLST sequence type	Pilus type	DEG analysis
								Sonicate fluid	Synovial fluid						
1	IDRL‐7463	2005	73	Male	Knee	4.5 years	33	∼	ND	None	Resection	II	22	2a	Not included
2	IDRL‐7656	2006	67	Male	Knee	11.6 years	107	∼	ND	Ceftriaxone	Resection	Ia	1651^a^	1, 2a	Included
3	IDRL‐8557	2009	69	Male	Knee	5.4 years	31	∼	ND	Cefadroxil, stopped 21 days before revision	Resection	Ia	23	2a	Included
4	IDRL‐9433	2012	55	Female	Knee	29 days	69	∼	ND	Clindamycin	Poly exchange	V	1	1, 2a	Included
5	IDRL‐10197	2015	42	Male	Hip	2 months	44	∼	∼	None	One‐stage exchange	V	1	1, 2a	Not included
6	IDRL‐11503	2016	63	Female	Hip	3 months	33	∼	∼	Levofloxacin	One‐stage exchange	V	1	1, 2a	Included

Abbreviations: DEG, differentially expressed gene; ND, not done.

^a^ Novel sequence type.

### 3.2 Genomic description of *S. agalactiae* isolates

**Figure A1:**
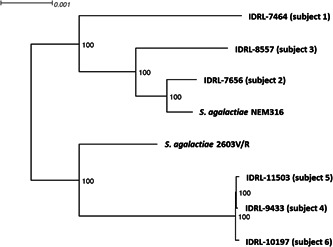
Phylogenic tree of the cultured isolates and *Streptococcus agalactiae* NEM316 and 2603V/R

Whole‐genome sequencing of the cultured isolates showed the isolates to have diverse characteristics. Three isolates displayed serotype V, two serotype Ia, and one serotype II (Table 1). There was also diversity in isolate multilocus sequence types (Table 1), with one isolate (IDRL‐7656/subject 2) displaying a novel sequence type due to a novel allele for *adhP* in the region used for typing. A phylogenetic analysis of the isolates, which included NEM316 (serotype III) and 2603V/R (serotype V) as references, recapitulated the diversity findings, with the serotype V isolates clustering together, and other isolates showing differences from one another (Figure A1). When screening for virulence genes against the VFDB database (Liu et al., 2019), it was shown that while the isolates had similar complements of virulence genes, they exhibited expected variation in the architecture of the capsular polysaccharide (CPS) genes corresponding to their respective serotypes. Fibrinogen‐binding surface protein genes *fbsA and fbsB* were found in IDRL‐7656 and IDRL‐8557. While the presence of pilus‐associated genes showed slight differences between the isolates, all genes belonging to *cyl* operon were detected in all isolates (Table S1, https://doi.org/10.5281/zenodo.5717630).

### 3.3 *S. agalactiae* expression profiles by outlier expression analysis

Total read counts of transcripts from sonicate fluids ranged from 31,291 to 522,023. Since Samples 1 and 4 had low read counts of non‐rRNA transcripts, they were excluded from expression analysis studies (Table A1). Read counts of non‐rRNA transcripts from *S. agalactiae* strain NEM316 RNA‐seq are shown in Table A2. The *S. agalactiae* pan‐genome constructed from the associated PJI isolates, comprised of 2738 genes, of which 1683 were identified as core genes, was used to quantify bacterial transcripts from PJI and the in vitro NEM316 strain. There were 227 genes identified as strong outliers (in vitro |*z*| > 3, where *z* is a modified *z* score—see Section 2) in expression between PJI (in vivo) and in vitro conditions. Of these, 164 were upregulated (in vitro *z* < −3), and 63 downregulated (in vitro *z* > 3) in sonicate fluid compared to in vitro. Genes with detected outlier expression are listed in Table S2 (https://doi.org/10.5281/zenodo.5717630) and whole core gene lists are shown in Table S3 (https://doi.org/10.5281/zenodo.5717630), identified by the matching locus tag in *S. agalactiae* 2603V/R used as an annotation reference.

**Figure A2:**
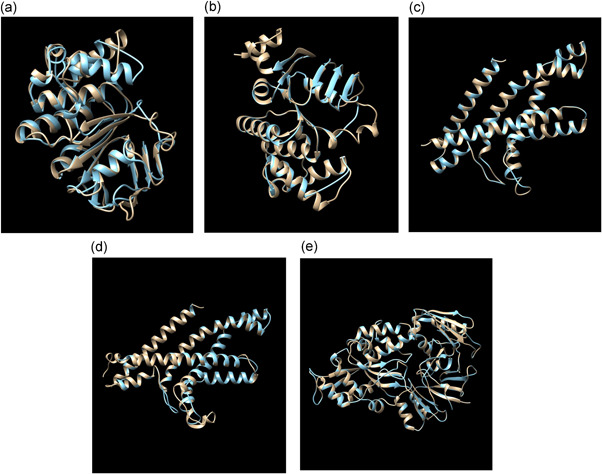
Protein structure homology model of *sag1514–1518* (a–e, respectively) produced by Phyre2. The predicted protein from *sag1514–1518* is shown in blue with the template protein (Table A3) shown in yellow

*nik* operon genes (*sag1514‐1518*), *cop* operon genes (*sag0384‐0386*), and *lmb‐phtD* operon genes (*sag1233‐1234*), encoding known or predicted metal transport systems, were highly expressed in sonicate fluid. *sag1514‐1518* comprise an operon putatively involved in intake of nickel/cobalt via *nikA–E* (ATP‐binding cassette [ABC] transporter), which are homologous with genes involved in a nickel/cobalt uptake system in *S. aureus*, *cntA‐D*, and *cntF*, which has been shown to contribute to *S. aureus* virulence (Remy et al., 2013). The function of these genes in *S. agalactiae* disease has not, however, been demonstrated. The predicted structure of the SAG1518 protein is homologous to the structure of NikA from *Brucella suis* (protein data bank ID: 4OER), with 93% coverage and 41% identity based on Phyre2 prediction (Kelley et al., 2015) and Chimera (Pettersen et al., 2004) alignment of the two structures (Table A3, Figure A2).

*sag0384‐0386* (*copR*, *copA*, and *copZ*) belonging to *cop* operon were highly expressed in sonicate fluid *versus* in vitro (*z* = −5.152, −16.313, and −1.386, respectively). *sag1264* encoding transcriptional repressor CopY was not detected in the in vitro strain.

*sag1234* encoding laminin‐binding protein (Lmb) and *sag1233* encoding streptococcal histidine triad family protein (PhtD or Sht), were highly expressed in sonicate fluid. The Lmb protein of *S. agalactiae*, also known as an adhesin that binds a human extracellular matrix component called laminin, is involved in zinc uptake (Moulin et al., 2016). Two Lmb homologs, AdcA (SAG0535) and AdcAII (SAG1938), redundant binding proteins that combine with the AdcCB translocon (SAG0155 and SAG0156) form a zinc‐ABC transporter, with their expression controlled by zinc‐dependent regulator AdcR (SAG0154) (Moulin et al., 2016). In this study, *sag0535* and *sag0154‐0156* were highly expressed in sonicate fluid compared to the in vitro strain, and *sag1938* was not detected in the in vitro strain.

### 3.4 Functional enrichment

**Figure 1:**
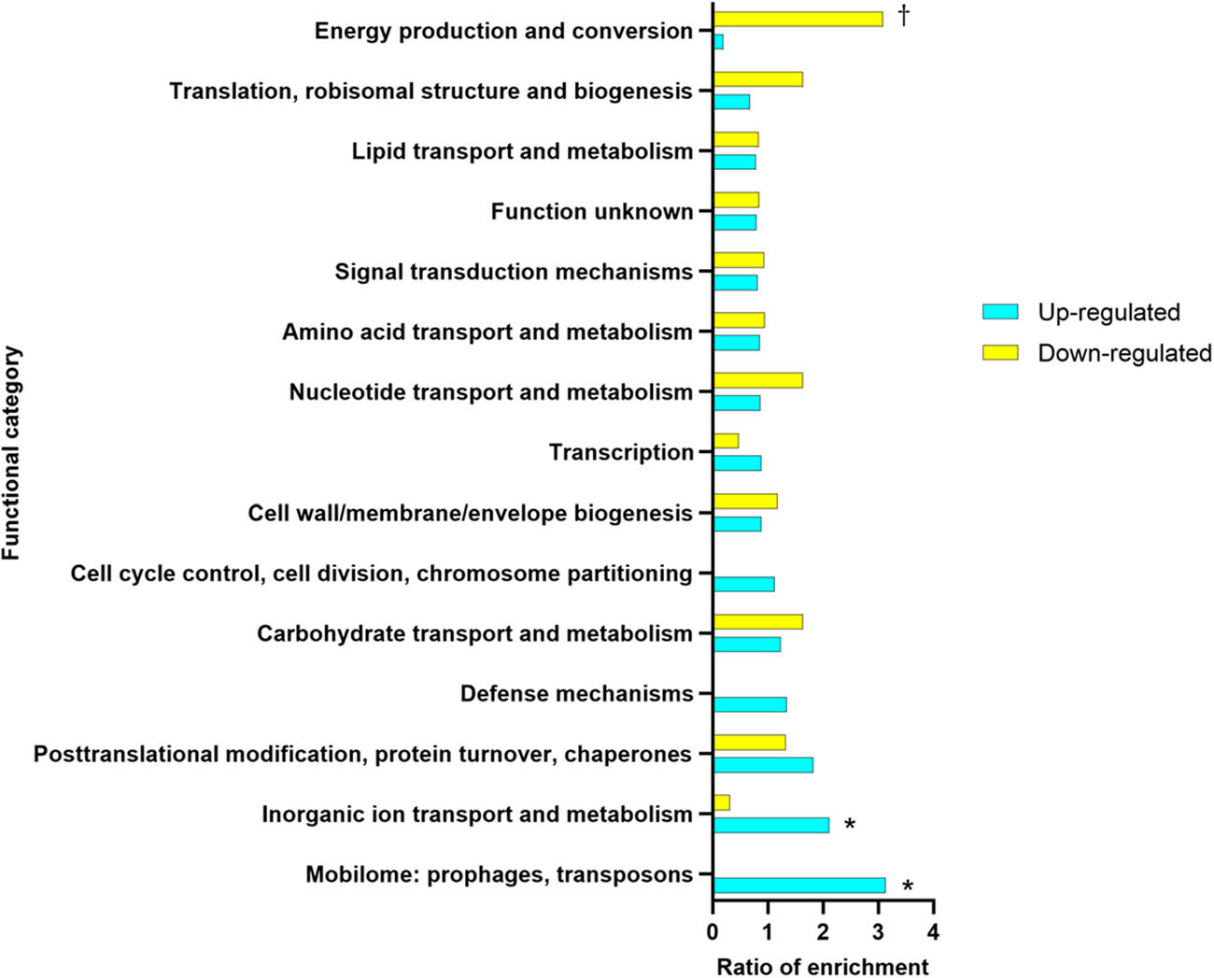
Functional enrichment of outlier *Streptococcus agalactiae* genes in periprosthetic joint infection sonicate fluid compared to NEM316 grown in vitro. Genes with |*z*| > 3 were considered outliers. The ratio of enrichment was calculated as the % of genes of a given functional category in the increased or decreased expressed RNA‐seq data set/% of genes assigned to the functional category in the *S. agalactiae* genome. Ribosomal protein, rRNA, and tRNA genes were removed. *Significant enrichment amongst genes increased; ^†^significant enrichment amongst genes decreased in sonicate fluid, with *p* < 0.05 (Fisher's exact test). rRNA, ribosomal RNA; tRNA, transfer RNA

Functional enrichment analysis of outlier genes revealed genes involved in mobilome and those in inorganic ion transport and metabolism to be most enriched in sonicate fluid (Figure 1). The most interesting genes in the outlier analysis, *nik*, *lmb‐phtD*, and *cop* operons, belonged to the inorganic ion transport and metabolism functional category. The pathogenic roles of the genes belonging to the mobilome, if any, are unknown.

Genes labeled as being involved in energy production and conversion based on the database of COGs (https://www.ncbi.nlm.nih.gov/research/cog) showed decreased expression in PJI *versus* in vitro, with the most downregulated genes in this category encoding F0F1 ATP synthase subunit C, alpha, gamma, and epsilon (*sag0857*, *sag0861*, *sag0862*, and *sag0864*, respectively).

### 3.5 Genes associated with *S. agalactiae* adhesion and biofilm formation

#### 3.5.1 Adhesion factors, pilus islands, and sortases

**Figure 2:**
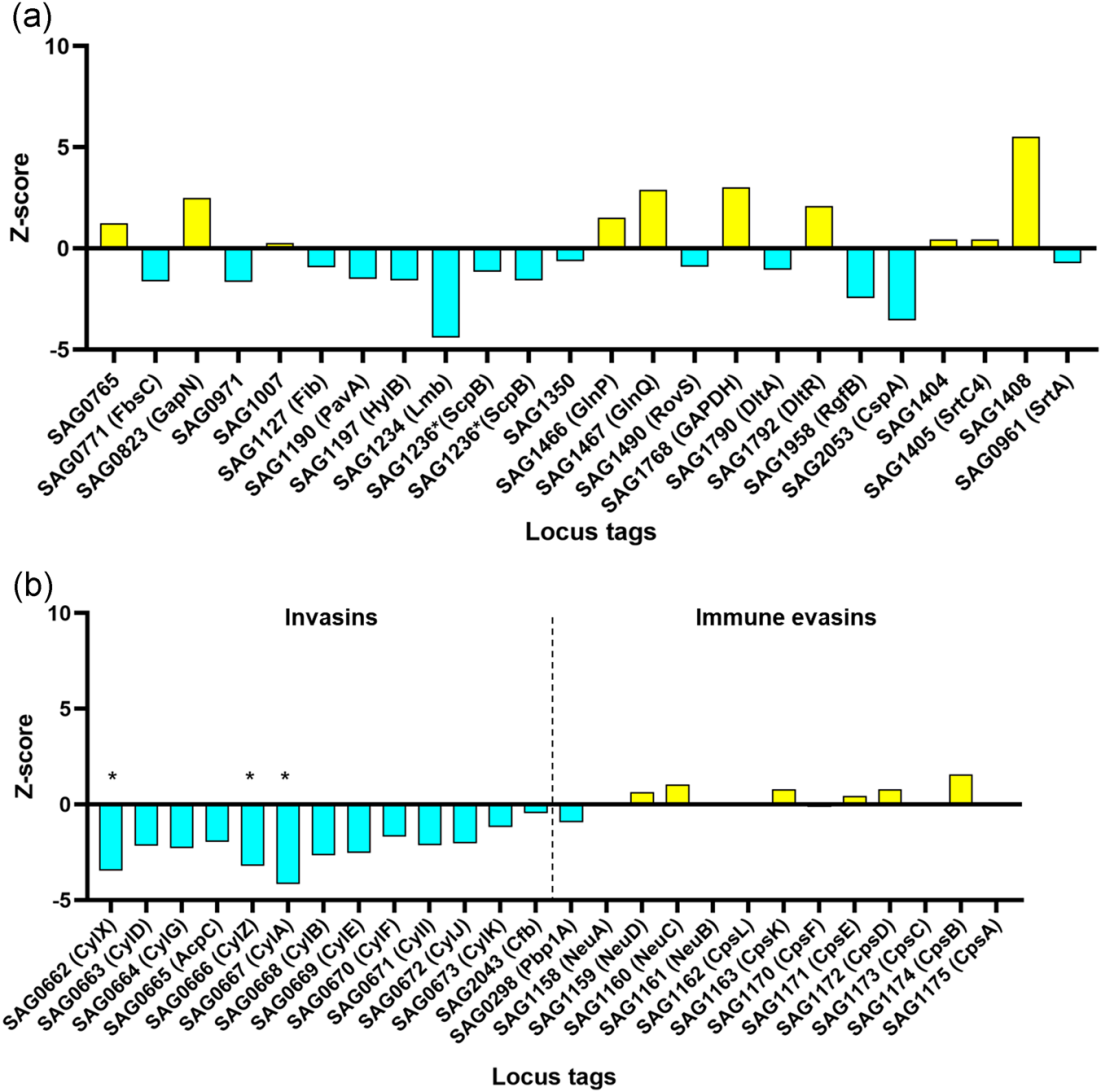
(a) Expression levels of genes involved in bacterial adhesion in sonicate fluid of four periprosthetic joint infection (PJI) subjects compared to NEM316 in vitro. *Upregulated outliers in sonicate fluid; ^†^downregulated outliers in sonicate fluid. (b) Expression levels of invasin and immune evasin genes in sonicate fluid of four PJI subjects compared to NEM316 in vitro. *Upregulated outliers in sonicate fluid; ^†^downregulated outliers in sonicate fluid

Expression of genes encoding adhesion factors important in *S. agalactiae* biofilm formation was analyzed (Table S4, https://doi.org/10.5281/zenodo.5717630); *lmb* and *cspA* were upregulated outliers, and *gap* downregulated in sonicate fluid compared to in vitro (Figure 2a). All isolates that caused PJI had PI‐2a, and four (including three of four isolates subjected to outlier expression analysis) had PI‐1, with *sag1408* encoding PI‐2a a downregulated outlier in sonicate fluid compared to in vitro. The gene encoding sortase, involved in cell wall anchoring of pilus polymers (Nobbs et al., 2008)—*srtA* (*sag0961*)—was weakly more expressed in sonicate fluid compared to in vitro (Table S4, https://doi.org/10.5281/zenodo.5717630, Figure 2a).

### 3.6 Other virulence factors

*cylA, B, D, E, F, G, I, J, K, X, Z*, and *acpC*, encoding β‐hemolysin/cytolysin were all upregulated in sonicate fluid compared to in vitro (Figure 2b). Among genes involved in immune evasion, *neuC‐D, cpsB, D, E*, and *cpsK* were downregulated in sonicate fluid compared to in vitro (Table S4, https://doi.org/10.5281/zenodo.5717630, Figure 2b).

## 4 DISCUSSION

*S. agalactiae* is a leading pathogen of invasive disease in neonates and pregnant women and also in nonpregnant adults, especially those of older age or with underlying conditions. Since *S. agalactiae* infections in newborns and pregnant women are known to start from bacterial colonization of the vagina, adhesion factors and other virulence factors associated with biofilm formation on the vaginal mucosa have been studied as contributors to colonization (Cook et al., 2018; Sheen et al., 2011). PJI is initiated through introduction of microorganisms at surgery, spread of infection from adjacent sites, or hematogenous seeding (Tande & Patel, 2014). Since bacteremia accompanies *S. agalactiae* PJI in up to 50% of cases, hematogenous spread is thought to be an important source (Everts et al., 2004). To establish infection, bacteria theoretically first colonize the gastrointestinal tract, genitourinary tract, and/or skin, where they form biofilms, and then spread hematogenously, adhere to prostheses, and again form biofilms on prosthesis surfaces. Studies on this complex pathological process are, however, limited.

In this study, the most enriched functional gene category in PJI sonicate fluid was the inorganic ion transport and metabolism category, of which genes involved in nickel, zinc, and copper transport were highly expressed. Metals play a role in life processes of microorganisms, with organisms having developed processes for their uptake. Pathogenic bacteria encounter metal restriction when placed in the metal‐poor environment of their host (Hammer & Skaar, 2012). “Nutritional immunity” set up by hosts to prevent bacterial growth presumably extends to many, if not all “essential” micronutrients, with mechanisms having been described for sequestering zinc, iron, and manganese (Grim et al., 2017; Kehl‐Fie & Skaar, 2010). The synovial space and surrounding tissues in which PJI occurs are limited spatially and in terms of nutrients (Jackson & Gu, 2009). The action of micrometallic molecules on surrounding human tissues, prostheses, and causative bacteria is an interesting topic; this study provides insight into this process. Although means of metal acquisition are well‐known for iron, manganese, and zinc (Corbin et al., 2008), others metals in trace amounts may be important under specific conditions (Remy et al., 2013). Nickel is a cofactor of bacterial enzymes potentially involved in a myriad of cellular processes (Mulrooney & Hausinger, 2003). For *Helicobacter pylori*, for example, nickel, a cofactor of urease, is essential for survival and successful colonization of human gastric mucosa (Molnar et al., 2010). Recently, a nickel/cobalt uptake system (CntA–D and F/NikA–E) in *S. aureus* has been shown to contribute to virulence of this species (Remy et al., 2013). In a murine bacteremia model, mortality was lower in *S. aureus cnt* mutant infection compared to wild‐type strain infection. Bladder and kidney colonization in a urinary tract infection model were reduced with the *cnt* mutant versus the wild‐type strain (Remy et al., 2013). In this study, *sag1514–1518* (*nikA–E*), genes putatively involved in nickel uptake, were highly expressed in sonicate fluid. Although the roles of these genes have not been demonstrated in *S. agalactiae*, gene orthology suggests that they may function similarly to the *S. aureus* CntA‐D and F system and play a role in PJI pathogenesis. The findings in *S. agalactiae* are novel and reported here for the first time. In addition, a transcriptome study revealed that *cnt* genes were upregulated in *S. aureus* PJI sonicate fluid compared to corresponding isolates grown in vitro (Le Masters et al., 2021). The finding of upregulation of *nik* and *cnt* genes shown in *S. aureus* and *S. agalactiae* PJI, respectively, suggests a potential role of nickel/cobalt uptake systems in the pathogenesis of PJI.

Copper is an essential metal element in bacterial cells. However, excessive copper is hazardous to cells due to free‐radical damage (Ladomersky & Petris, 2015). Keeping a balance of copper at human pathogen interfaces is needed for bacterial survival and pathogenesis. *copA* encoding the copper‐transporter ATPase CopA mediates control of copper efflux in several human pathogens (Johnson et al., 2015; Ladomersky et al., 2017; Macomber & Imlay, 2009; White et al., 2009). A recent study showed the role of this mechanism on survival, growth, and virulence of *S. agalactiae* in the mammalian host (Sullivan et al., 2021). Although copper levels in sonicate fluid were not determined in this study, they are known to be elevated in inflamed tissue (Djoko et al., 2015). As copper levels in infected tissues are increased, this may be related to increased expression of *cop* operon genes, which regulate copper efflux for virulence and survival of bacteria, in sonicate fluid.

Among the virulence factors studied, *lmb* and *csp*A were highly expressed in PJI compared to in vitro. Lmb is an adhesin that binds to laminin in human tissue; it also promotes bacterial invasion in human brain microvascular endothelial cells (Spellerberg et al., 1999; Tenenbaum et al., 2007). Lmb is also involved in zinc uptake, showing homology with the zinc‐binding protein AdcA of other streptococcal species (Bayle et al., 2011; Linke et al., 2009). Zinc is also a trace element that serves as a cofactor for a number of essential prokaryotic enzymes and transcriptional regulators. Pathogenic bacteria must adapt zinc transport mechanisms to accommodate these differences to both avoid toxicity and meet their requirements for this metal. In a zinc‐deficient environment, zinc acquisition in streptococci is mostly performed by an ABC transporter, which is composed of one or several metal‐binding proteins (AdcA, Lbp, or Lmb), an integral membrane component (AdcB), and an ATPase (AdcC) (Moulin et al., 2016). In contrast, in the presence of adequate intracellular zinc concentrations, the AdcR repressor regulator inhibits expression of *adcABC* and *lmb*. In this study, these zinc uptake genes, *adcABC*, and *lmb* were highly expressed in sonicate fluid compared to in vitro, suggesting a potential role of increased zinc uptake in the pathogenesis of *S. agalactiae* PJI.

Although biofilm formation by *S. agalactiae* may be associated with PI‐2a pilus production (Rinaudo et al., 2010), expression of PI‐2a pilus genes was downregulated in sonicate fluid compared to in vitro in this study. While some studies have suggested that nonpilus adhesion regulated by *covR* may be a contributor to bacterial adherence and biofilm formation (Park et al., 2012), *covR* (*sag0416*) expression was only weakly higher in sonicate fluid compared to in vitro conditions in this study. This suggests that biofilms formed on arthroplasty surfaces may be affected by the expression of nonpilus rather than pilus adhesins, or other mechanisms.

β‐Hemolysin/cytolysin (β‐HC, also CylE), is a surface‐associated, pluripotent toxin crucial for *S. agalactiae* pathogenesis; it promotes *S. agalactiae* invasion of lung epithelial and endothelial cells and the blood–brain barrier (Rajagopal, 2009). Hemolytic activity is associated with *S. agalactiae* colonization and pathogenesis, with hemolysin‐deficient *S. agalactiae* mutants being attenuated for virulence in a *S. agalactiae* arthritis murine model; while more joint inflammation and damage were observed with hyperhemolytic mutant‐infected animals than in those infected with the parental strain, nonhaemolytic mutant‐infected mice had mild and transient arthritis (Puliti et al., 2000). The *cyl* operon (*cylX‐K*) is necessary for the synthesis of granadaene, the ornithine rhamnolipid pigment in *S. agalactiae*, which is hemolytic and cytotoxic to human amniotic epithelial cells and innate immune cells (Armistead et al., 2020; Forquin et al., 2007; Gottschalk et al., 2006; Rosa‐Fraile et al., 2014; Whidbey et al., 2013). In this study, all 12 genes belonging to *cyl* operon were highly expressed in PJI compared to in vitro, suggesting that they could contribute to the pathogenesis of PJI.

*S. agalactiae* is encapsulated by a sialic acid CPS. Since sialic acid is also present on glycans of eukaryotic cells, the host may not recognize *S. agalactiae* as nonself (Rajagopal, 2009). Accordingly, CPS prevents complement factor C3 deposition and phagocytosis of *S. agalactiae* (Rajagopal, 2009). The genes required for CPS synthesis are part of a single *cps* locus, harboring a variable serotype‐determining region (*cpsG–cpsK*) flanked by other CPS genes (*cpsA*–*cpsF* and *neuB*–*neuA*) conserved among different serotypes (Cieslewicz et al., 2005). In this study, expression of *cpsB*, *D*, *E*, *G*, and *K*, *neuC*, and *D* was downregulated in PJI compared to in vitro, although not among outliers. Contrary to a recent study that reported that *cps* genes are conditionally essential for the survival of *S. agalactiae* in human blood (Hooven et al., 2017), the role of *cps* genes in PJI might be less significant.

There are several limitations to this study. Ideally, in vitro transcriptomic analysis of each isolate under conditions corresponding to each sample in vivo would have been helpful to understand the potential pathogenic role of the genes analyzed. NEM316 and the conditions under which it was grown in vitro may not be representative of the whole *S. agalactiae* population or the PJI isolates studied. That said, NEM316 is a human strain from invasive disease and biofilm‐producing, which is also relevant to PJI. In this study, functional validation of novel genes identified was not performed. Finally, gene expression may have been affected at least in part by the specimen processing used. The lack of an RNA stabilizer is also a limitation.

In conclusion, the data generated provides a glimpse into the transcriptomic landscape of *S. agalactiae* in the environment around prosthetic joints. Using outlier expression and functional enrichment analysis, the *nik* operon was upregulated in PJI, suggesting a role of nickel transport in PJI pathogenesis. Among known virulence factors, β‐HC was consistently upregulated in PJI. The findings presented contribute to understanding of *S. agalactiae* PJI pathogenesis and provide molecular targets for the identification of novel PJI therapeutics or future vaccines against invasive infections caused by *S. agalactiae*.

## CONFLICT OF INTERESTS

Dr. Patel reports grants from ContraFect, TenNor Therapeutics Ltd, Hylomorph, BioFire, and Shionogi. Dr. Patel is a consultant to Curetis, Specific Technologies, Next Gen Diagnostics, PathoQuest, Selux Diagnostics, 1928 Diagnostics, PhAST, Torus, Mammoth Biosciences, and Qvella; monies are paid to Mayo Clinic. Dr. Patel is also a consultant to Netflix. In addition, Dr. Patel has a patent on *Bordetella pertussis/parapertussis* PCR issued, a patent on an anti‐biofilm substance issued, and a patent on a device/method for sonication with royalties paid by Samsung to Mayo Clinic. Dr. Patel receives an editor's stipend from the Infectious Diseases Society of America, and honoraria from the National Board of Medical Examiners, the Infectious Diseases Board Review Course, and UpToDate Inc. All other authors declare no conflict of interests.

## ETHICS STATEMENT

This study was performed under approval from Mayo Clinic Institutional Review Board (Protocol No. 09‐000808).

## AUTHOR CONTRIBUTIONS

**Hye‐Kyung Cho**: Conceptualization (supporting), formal analysis (lead), writing – original draft (lead). **Thao Masters**: Conceptualization (supporting), Investigation (supporting), writing – review and editing (supporting). **Kerryl E. Greenwood‐Quaintance**: Supervision (lead), writing – review and editing (supporting). **Stephen Johnson**: Data curation (supporting), writing – review and editing (supporting). **Patricio R. Jeraldo**: Data curation (lead), writing – review and editing (supporting). **Nicholas Chia**: Validation (supporting), writing – review and editing (supporting). **Meng Pu**: Investigation (supporting), writing – review and editing (supporting). **Matthew P. Abdel**: Validation (supporting), writing – review and editing (supporting). **Robin Patel**: Conceptualization (lead), writing – review and editing (lead).

## Data Availability

All data generated or analyzed during this study are included in this published article (and its Supporting Information Files). New sequence data from this study have been deposited into NCBI under project no. PRJNA687554: https://www.ncbi.nlm.nih.gov/bioproject/PRJNA687554 (BioSample accessions: SAMN17305169‐SAMN17305174). Tables S1–S4 have been deposited on Zenodo (https://doi.org/10.5281/zenodo.5717630).

## 1

**Table A1:** RNA counts from sonicate fluid of *Streptococcus agalactiae* PJI subjects (in vivo)

S. no.	No. of predicted genes	No. of protein‐coding genes	Total reads counts of quantified transcripts	Read counts of non‐rRNA transcripts	% Non‐rRNA reads	DEG analysis
1	2031	1957	56,989	3483	6.1	Not included
2	2240	2166	175,647	88,497	50.4	Included
3	2062	1983	324,455	283,023	87.2	Included
4	2145	2074	522,023	483,179	92.6	Included
5	2095	2020	31,291	1849	5.9	Not included
6	2080	2008	459,667	252,168	54.9	Included

Abbreviations: DEG, differentially expressed gene; PJI, periprosthetic joint infection; rRNA, ribosomal RNA.

**Table A2:** RNA counts from *Streptococcus agalactiae* strain NEM316 RNA‐seq (in vitro)

S. no.	Read counts of non‐rRNA transcripts
1	1,530,792
2	1,708,503
3	1,224,999

Abbreviation: rRNA, ribosomal RNA.

**Table A3:** Confidence, coverage, and identity values of predicted nickel transport genes matched to each template in the Phyre2 model

Old locus tag	Gene name	Template PDB code	PDB title	Aligned residues	Alignment coverage (%)	Confidence (%)	Identity (%)
*sag1514*	*nikE, cntF*	4FWI	Crystal structure of the nucleotide‐binding domain of a dipeptide ABC transporter	1–215	94	100	29
*sag1515*	*nikD, cntD*	4FWI	Crystal structure of the nucleotide‐binding domain of a dipeptide ABC transporter	3–252	95	100	32
*sag1516*	*nikC, cntC*	4YMU	Crystal structure of an amino acid ABC transporter complex with arginines and ATPs	56–262	76	99.9	13
*sag1517*	*nikB, cntB*	4YMU	Crystal structure of an amino acid ABC transporter complex with arginines and ATPs	82–309	72	99.9	15
*sag1518*	*nikA, cntA*	4OER	Crystal structure of NikA from *Brucella suis*, unliganded form	32–533	93	100	41

Abbreviation: PDB, protein data bank.
